# The Impact of 5-Hydroxytryptophan Supplementation on Cognitive Function and Mood in Singapore Older Adults: A Randomized Controlled Trial

**DOI:** 10.3390/nu17172773

**Published:** 2025-08-27

**Authors:** Shuqi Li, Clarinda Nataria Sutanto, Xuejuan Xia, Jung Eun Kim

**Affiliations:** 1 Department of Food & Technology, National University of Singapore, Singapore 117542, Singapore; e1124672@u.nus.edu (S.L.); cnsfst@nus.edu.sg (C.N.S.); xiaxj@usst.edu.cn (X.X.); 2 Bezos Center for Sustainable Protein, National University of Singapore, Singapore 117542, Singapore

**Keywords:** 5-HTP, cognitive function, mood, serotonin

## Abstract

**Objectives:** Concurrent with global aging epidemics, cognitive decline has become an increasing public health concern. Dietary supplementation may offer neuroprotective benefits, and 5-hydroxytryptophan (5-HTP) has gained interest due to its role in serotonin synthesis, thereby regulating cognitive function and mood. However, there is limited evidence on its effect on cognitive function, especially among older Asian adults. Therefore, the study aimed to investigate the effects of 5-HTP supplementation on cognitive function and mood in Singaporean older adults. **Methods:** This was a single-blinded, 12-week randomized controlled trial, and 30 participants (66 ± 3 years) were randomly assigned to consume 100 mg of 5-HTP daily or not consume it. Cognitive function and mood were assessed via the Montreal Cognitive Assessment (MoCA), Geriatric Anxiety Inventory (GAI), and Geriatric Depression Scale (GDS). Cognitive function-related blood biomarkers, including amyloid beta (Aβ)40, Aβ42, gamma-aminobutyric acid, and serotonin, were also determined. **Results:** A significant time effect was observed in the MoCA score, which was mainly explained by a significant increase in the 5-HTP group (week 0 vs. week 12: 26.6 ± 1.4 a.u. vs. 27.6 ± 1.4 a.u., *p* < 0.05). Moreover, the 5-HTP group showed a significant increase in serum serotonin levels. Additionally, the GDS score improved in the 5-HTP group (week 0 vs. week 8: 1.2 ± 1.7 a.u. vs. 0.7 ± 1.2 a.u., *p* < 0.05). However, no effects on GAI and other biomarkers were observed. **Conclusions:** 5-HTP supplementation can enhance cognitive performance and reduce symptoms of depression in Singaporean older adults, potentially through serotonergic modulation. However, given the relatively small sample size (*n* = 30) and short-term (12-week) intervention, these findings should be interpreted cautiously, and further long-term studies with a larger sample size are warranted to confirm these preliminary results.

## 1. Introduction

Population aging is a global phenomenon, as the average life expectancy has significantly extended due to improvements in healthcare [1]. Singapore’s population is aging rapidly, similar to that of many developed countries, and the proportion of individuals aged 65 years and older is expected to reach 25% by 2030 [2]. Although aging may not be a direct cause of dementia, a neurodegenerative disorder with a decline in cognitive function [3], it is a vital non-modifiable risk factor for the development of dementia and the population with dementia in Singapore increased by approximately 42% from 2013 to 2023 [4]. The decline in cognitive function is also associated with depression and anxiety [5], and individuals with dementia have been reported to experience significantly higher rates of depressive and anxiety symptoms compared to cognitively healthy older adults [6].

Currently, no pharmaceutical therapy can reverse the progression of dementia; therefore, more focus has been placed on lifestyle-based prevention strategies, such as following a healthy diet, increasing physical activity, and even performing cognitive training [7,8]. Among these, dietary interventions, including taking dietary supplementation, have gained increasing interest for their potential to support brain health, as accumulating evidence indicates that nutritional factors play an essential part in regulating brain aging and neuroprotection [9,10,11]. In addition, several studies have reported that taking specific dietary supplements are associated with improvements in mood, especially in reducing symptoms of depression and anxiety in older adults [12,13].

Particularly, the regulation of the serotonergic system via dietary modification has attracted attention due to its role in regulating mood, cognition, neuroplasticity, and overall brain health [9,14]. Serotonin, a key neurotransmitter, influences a wide array of physiological processes such as sleep, body temperature, appetite, and motor activity [15], as well as psychological processes, including memory, learning, and executive function [16]. A recent prospective study found that a lower serum serotonin level is linked with increased risks of functional decline, mild cognitive impairment, and brain atrophy [17]. Age-related declines in serotonin levels and serotonergic function have been linked with reduced prefrontal cortex activity and compensatory shifts in brain activation patterns during cognitive tasks [14].

5-Hydroxytryptophan (5-HTP), a natural amino acid, has been proposed as a potential dietary supplement to enhance central serotonin synthesis. 5-HTP is a direct metabolic intermediate in the biosynthesis of serotonin by the enzyme aromatic L-amino acid decarboxylase in the central nervous system. 5-HTP supplementation has been shown to enhance brain serotonin levels in previous non-human primate studies [18,19]. A recent systematic review and meta-analysis of clinical trials found that 5-HTP supplementation significantly increased total plasma levels of serotonin and its metabolites in adults and children with cognitive or mood disorders [20]. Some clinical studies have reported beneficial effects of 5-HTP in treating depression [21] and improving sleep quality [22].

However, the evidence on its role in cognitive enhancement remains limited and inconclusive. Despite the biological plausibility and preliminary evidence, clinical studies that have directly assessed the effect of 5-HTP supplementation on cognitive function are lacking. This gap is particularly pronounced in older adults who are generally experiencing cognitive decline and showing a higher risk of mental distress and neurodegenerative disorders. Additionally, given the growing burden of cognitive decline in Singapore’s aging population [4], there is a compelling need to explore promising supplementation interventions in this population. Hence, to address these gaps, this study aimed to evaluate the effects of 5-HTP supplementation on cognitive function and mood in Singaporean older adults through a 12-week, randomized, controlled trial (RCT). Validated questionnaires and blood biomarkers were employed to assess outcomes. It was hypothesized that 5-HTP supplementation could improve cognitive function and mood through the regulation of the serotonergic system in older adults.

## 2. Methods

### 2.1. Participant

**Figure 1:**
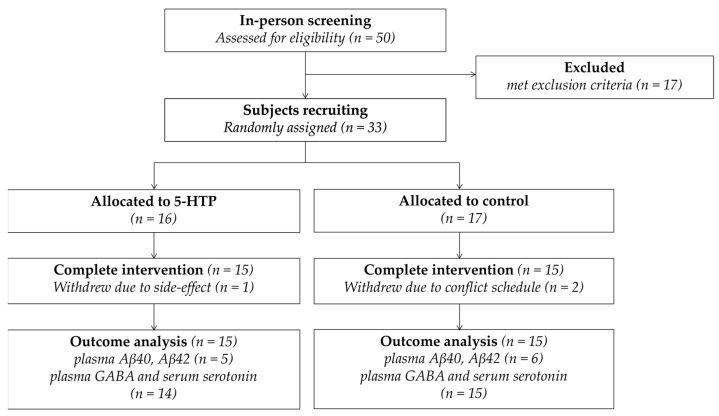
CONSORT flow chart.

The participants were Singaporean adults, aged between 60 and 85 years. They were recruited primarily through word-of-mouth between July 2020 and March 2021. Individuals from a pool of previous study participants were invited, and they were drawn from various regions across Singapore. Inclusion criteria included being able to give informed consent, being aged between 60 and 85 years, and being literate in English. Exclusion criteria included having lost more than 3 kg of weight within the past 3 months; having engaged in vigorous exercise within the past 3 months; having consumed 5-HTP, serotonin, or tryptophan supplements for the past month; currently being diagnosed with acute illnesses; taking medication for hypertension, hypercholesterolemia, or diabetes for less than 5 years; smoking; having more than 2 alcoholic drinks per day; and having insufficient venous access for blood draw. Interested participants were scheduled for a phone screening to assess initial eligibility. This was then followed by an in-person screening session. During the screening, the details about the study were explained, and written informed consent was obtained. Eligible participants were then scheduled for a subsequent study visit, during which baseline data were collected and randomization was carried out. The sample size was determined based on a prior power analysis, as described in detail in Section 2.8. A total of 50 participants were screened in-person, of whom 33 were recruited for the study. By June 2021, 30 participants had completed the trial, 15 in the 5-HTP group and 15 in the control group. Among them, 1 participant withdrew due to gaining an increased appetite after the daily consumption of 5-HTP, while 2 participants withdrew due to scheduling conflicts. Reimbursement was provided to participants for their participation and completion of the study. All in-person screenings, study visits, and data collection were conducted at the National University of Singapore (NUS). Data were collected and analyzed from a total of 30 participants, except for amyloid beta (Aβ)40, Aβ42 (n = 11), gamma-aminobutyric acid (GABA), and serotonin (n = 29). The Consolidated Standards of Reporting Trials (CONSORT) flow diagram is illustrated in Figure 1.

### 2.2. Study Design

This was a 12-week, parallel, single-blinded RCT, and participants were either randomized into the intervention group (5-HTP) to consume 100 mg per day of 5-HTP capsules (NOW Foods, Bloomingdale, IL, USA) 20–30 min before bedtime or the control group (Control), not to consume 5-HTP. This daily dosage of 5-HTP was selected based on the previous clinical studies, where 50–200 mg were administered and demonstrated safety and efficacy in terms of body composition management and mental health regulation [21,23,24,25]. Given that the participants in this study were cognitively healthy and had not previously taken 5-HTP supplementation, 100 mg was selected to balance the potential benefits with minimal risk of adverse events. Randomization was generated using STATA version 14 (StataCorp LP, College Station, TX, USA) and conducted by an independent member outside the study team. The main researchers were blinded to the randomization before completing the outcome measurements and data analysis.

**Figure 2:**
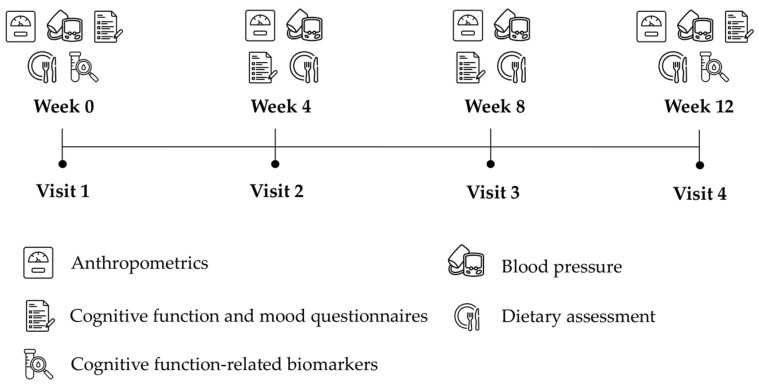
Study design.

Participants were expected to complete 4 visits at 4-week intervals; the details of the measurements are shown in Figure 2. Anthropometrics, blood pressure, cognitive function and mood, and dietary intake were assessed at every visit, and blood was collected at weeks 0 and 12 to measure cognitive function-related biomarkers. This RCT was registered in clinicaltrials.gov (NCT04078724) with the approval of the National University of Singapore Institutional Review Board (NUS-IRB-H-19-037).

### 2.3. Anthropometric Parameters and Blood Pressure Measurements

The height and weight of participants were measured twice by a stadiometer (BSM370, InBody, Seoul, Republic of Korea), and BMI was calculated based on the average of the two measures. The waist circumference was measured twice using a measuring tape under guidelines from the World Health Organization [26]. Blood pressure was recorded twice by a blood pressure monitor (HEM-7121, Omron, Kyoto, Japan).

### 2.4. Dietary Assessment

During the 12-week intervention, all participants were required to maintain their habitual diet. The self-recorded dietary data were collected using a 3-day food record, which included 2 weekdays and 1 weekend to estimate their daily average consumption. The dietary data were then analyzed by DietPlan 7 (Forestfield Software Ltd., Horsham, UK) with nutritional information from the USDA database [27], supplemented with the “Energy & Nutrient Composition of Food” database from the Health Promotion Board of Singapore for local dishes [28], as well as the available nutrition labels of commercial food products.

### 2.5. Cognitive Function Assessment

The primary outcome of cognitive function was assessed by the Montreal Cognitive Assessment (MoCA), a widely used cognitive function test, including several cognitive domains (attention and concentration, executive functions, memory, language, visuoconstructional skills, conceptual thinking, calculations, and orientation) to detect mild cognitive dysfunction [29]. MoCA has demonstrated good internal consistency and high test–retest reliability in older adults, based on previous studies in Singapore [30,31]. The total score ranges from 0 to 30, with a higher score indicating better cognitive function, and a score of ≥26 is considered normal. The test was administered by trained researchers, and it took approximately 10 min to administer [32].

### 2.6. Mood Assessments

The Geriatric Depression Scale (GDS) and Geriatric Anxiety Inventory (GAI) are common screening tools used in clinical research studies to assess depressive or anxiety symptoms, respectively [33]. GDS and GAI are designed especially for the older population, minimizing the influence of physical illnesses by including a few somatic items, and employing only yes/no and agree/disagree formats to make them easier to administer [34,35]. Participants were instructed to complete both the GDS and GAI based on how they felt over the preceding week. Both GDS and GAI have shown strong validity and reliability in the Asian population across different ages, genders, and ethnicities [36,37]. GDS consists of 15-item self-reported measures of depressive symptoms; the answers yes/no were scored 1/0, respectively, with a higher score indicating more severe depression, and a score ≥ 5 indicating clinically relevant depressive symptoms [38]. GAI consists of a 20-item self-reported measure of anxiety symptoms, where the answers agree/disagree were scored 1/0, respectively, with a higher score indicating a higher anxiety status, and a score ≥ 11 suggesting generalized anxiety disorder [39].

### 2.7. Blood Sample Preparation and Analysis

Fasting blood (10 h) samples were collected by trained phlebotomists, and plasma (treated by EDTA vacutainers) and serum (treated by plain vacutainers) samples were obtained after sitting for 30 min at room temperature and were centrifuged at 3000× *g* for 15 min at 4 °C (Eppendorf, Leipzig, Germany). Samples were then stored in aliquots at −80 °C before analysis. Cognitive function-related biomarkers, including serum serotonin (Abcam, ab133053, Cambridge, UK), plasma Aβ40 and Aβ42 (G-Biosciences, 786-437, St. Louis, MO, USA), and plasma GABA (Wuhan Fine Biotech Co., Ltd., EH3098, Wuhan, China), were all determined using enzyme-linked immunoassay kits.

### 2.8. Power Calculation and Data Analysis

A power calculation was conducted a priori (G*Power 3.1, Heinrich Heine University, Düsseldorf, Germany) based on a previous preclinical study, which assessed the acute response in blood serotonin levels after administering 20 mg/kg of 5-HTP. Administration of 5-HTP showed significantly higher blood serotonin levels compared to without 5-HTP administration (55 ± 3 pg/mL vs. 20 ± 8 pg/mL, mean ± SEM) in non-human primates [19]. Assuming a similar response as previously, a sample size of 5 participants per group would be required to detect a statistically significant difference at 95% power and a significance level of 0.05 (two-tailed).

The 4-parametric logistic (4PL) method was used to calculate the concentrations of blood parameters related to cognitive function. Independent t-tests were performed to examine between-group differences after a normality test. Two-way repeated-measures analysis of variance (ANOVA) was used to analyze the main effects of time and group-by-time interactions on dependent variables, and Tukey’s multiple comparisons test was used to examine within-group differences between baseline and various post-intervention time points. All analyses were conducted by GraphPad Prism 9.0 (GraphPad Software Inc., San Diego, CA, USA). Results were presented as mean ± standard deviation (SD), and statistical significance was defined as *p* < 0.05 (two-tailed).

## 3. Results

### 3.1. Baseline Characteristics

The baseline characteristics, social demographics, and medical conditions of the 5-HTP group and the control group are shown in Table 1. The mean age of each group was 66 ± 3 and 67 ± 4 years, respectively, and both groups showed a good balance between female and male participant distribution. Regarding anthropometric parameters and blood pressure, there were no differences between the 5-HTP group and the control group at baseline.

### 3.2. Dietary Assessments

Nutrient intake is shown in Supplementary Table S1. There were no differences in energy, macronutrient, and micronutrient intake between the 5-HTP group and the control group at every visit.

### 3.3. Effect of 5-HTP Supplementation on Cognitive Function

The MoCA results are shown in Table 2. There was no difference in the MoCA total and individual scores between the 5-HTP group and the control group at week 0. A significant time effect (*p* = 0.0007) was observed in the MoCA total score, which was particularly explained by a significant increase in the 5-HTP group (week 0 vs. week 12: 26.6 ± 1.4 a.u. vs. 27.6 ± 1.4 a.u., *p* < 0.05). Additionally, significant time effects were also observed in naming (*p* < 0.0001), language (*p* = 0.0003), and abstraction (*p* = 0.007).

### 3.4. Effect of 5-HTP Supplementation on Cognitive Function-Related Biomarkers

The levels of cognitive function-related biomarkers are shown in Table 3. There were no differences in these biomarkers between the 5-HTP group and the control group at week 0. A significant group-by-time interaction effect (*p* = 0.0197) was observed in the serum serotonin level, and this result was particularly explained by a significant increase in the 5-HTP group (week 0 vs. week 12: 173.7 ± 81.2 ng/mL vs. 219.6 ± 73.1 ng/mL, *p* < 0.05). Also, a significant difference was observed in the change in the serum serotonin level from week 12 to week 0 between the two groups (5-HTP vs. Control: 45.8 ± 64.8 ng/mL vs. −20.2 ± 75.3 ng/mL, *p* < 0.05). However, no effects were observed in the levels of plasma Aβ40, Aβ42, and GABA, or in the Aβ42/Aβ40 ratio.

### 3.5. Effect of 5-HTP Supplementation on Mood

The GDS and GAI results are shown in Table 4. The GDS score significantly decreased at week 8 in the 5-HTP group compared to week 0 (week 0 vs. week 8: 1.2 ± 1.7 a.u. vs. 0.7 ± 1.2 a.u., *p* < 0.05), while 5-HTP supplementation did not affect the GAI score.

## 4. Discussion

During the process of aging, people generally encounter the deterioration of cognitive function, including the decline in memory, executive function, processing speed, and verbal abilities [40]. Non-pharmaceutical interventions, including dietary intervention, are gaining attention in improving cognitive function and mental health in this population, and certain amino acids such as tryptophan and 5-HTP have been investigated since they play a significant role in the synthesis of serotonin, which is involved in the regulation of cognitive function, mood, and other physiological activities [20,41]. Particularly, compared to tryptophan, 5-HTP can bypass the rate-limiting hydroxylation step, which makes it more efficient as a serotonin precursor [18,42]. In this study, we found that daily consumption of 100 mg 5-HTP improved cognitive function and mood, accompanied by a higher level of serum serotonin in Singaporean older adults.

Overall improvement in cognitive function was observed in both groups; only the 5-HTP group exhibited a statistically significant improvement from baseline. In this study, the mean increase in MoCA score in the 5-HTP group was approximately 1 point; however, a previous study indicated that a change of >5 points in the MoCA score can truly reflect an improvement in cognitive function [43]. A previous cohort study evaluated the minimal clinically important difference (MCID) for MoCA score in stroke survivors, and it is reported that MCIDs ranging from 1 to 1.6 points are associated with improvement in daily function and the quality of life [43]. Although the clinical meaning of a 1-point increase in healthy older adults has not been fully evaluated, this MCID range in stroke patients may provide a useful reference. However, caution is warranted in the interpretation of our results. Nonetheless, this improvement may be attributed to the enhanced serotonergic signaling in the central nervous system (CNS). A significant increase in the serum serotonin level supports this mechanism, as 5-HTP could cross the blood-brain barrier and synthesize serotonin, thereby effectively elevating serotonin levels and increasing central serotonergic tone [44]. Serotonin plays a key role in regulating cognitive functions, such as learning, memory, and executive function, particularly in the prefrontal cortex [16]. An overall increase in MoCA total score can reflect improvements in these domains, possibly mediated by enhanced serotonergic signaling in frontal regions. Although the serum level of serotonin did not fully reflect the central level, a previous study has shown that peripheral serotonin may partially reflect the changes in the CNS [45]. Across domains, significant time effects were shown in the domains of naming, language, and abstraction. Naming depends on semantic memory [46], and bilateral occipital lobes are commonly activated [47]. Language, engaging both temporal and frontal regions, is mainly supported by frontal lobe function [46]. In abstraction, frontal executive function is particularly needed. This aligns with previous studies indicating the modulatory role of serotonin in executive function and language processing [48,49]. Although statistical significance was only observed in the 5-HTP group, the control group also showed improvement to a certain degree; thus, caution is warranted in the interpretation of results, and further investigation into other factors that may affect cognitive function is required in both groups.

Although there was a significant increase in the serotonin levels after 5-HTP supplementation, no significant changes in other cognitive function-related biomarkers were observed. An accumulation of soluble Aβ40 and Aβ42 in the brain is a core neuropathological characteristic of Alzheimer’s disease (AD), and Aβ40 and Aβ42 are primarily relevant in the population at risk for or diagnosed with AD [50]. However, our participants were cognitively healthy, and their plasma Aβ40 and Aβ42 levels were consistent with a prior study, which reported that plasma Aβ levels were about 10 pg/mL in the non-demented population [51]. The variability observed in plasma Aβ levels, reflected by the large standard deviations, may be attributed to the inherent individual differences in circulating Aβ levels among older adults and the small sample size (n = 11), which may amplify data dispersion. Despite using validated ELISA kits, plasma Aβ levels are known to fluctuate and can be influenced by multiple biological factors [52,53]. GABA exerts an inhibitory effect through specific receptors in the CNS, providing phasic and tonic inhibition, thus controlling neuronal activity and supporting normal cognitive function in cortical circuits [54]. One of the receptors serotonin acts on is 5-Hydroxytryptamine-1A (5-HT_1_A), which is located on GABAergic interneurons [55]. Previous animal studies found that when serotonin binds to 5-HT_1_A receptors, GABAergic tone is strengthened, and it enhances the release of GABA [56,57]. However, receptor adaptation in the brain may happen with no change in plasma GABA levels [58].

Noticeably, although statistically not significant, the overall GDS score decreased (*p_time_* = 0.0564), with a significant decrease in week 8 only in the 5-HTP group, suggesting a potential benefit of 5-HTP in reducing depression. A study that treated Parkinson’s disease patients with 5-HTP showed improvement in depressive symptoms after 4 weeks of intervention [21]. Prior studies have also observed the effect of 5-HTP on mood regulation, especially improvements in patients with depression [21,59], as a deficiency of cerebral serotonin is one acknowledged possible cause of depression [60]. Thus, an increase in serotonin in 5-HTP supplementation may explain the improvement of depression symptoms. However, no change in GAI was observed, and this divergent response may be attributed to the different roles of neurobiological systems in depression and anxiety disorders. Although both are involved in serotonin, norepinephrine, dopamine, and GABA systems [61,62], GABAergic tone plays a more critical role in anxiety, while serotonin has a more well-established role in depression [60,63].

The main strength of this study lies in its contribution to the understanding of the effect of 5-HTP supplementation on cognitive function in older Asian adults. There is a paucity of data in older Asian adults, and moreover, previous studies have focused on mood, sleep, and appetite outcomes, particularly in the Western population [18,25,64]. Although the participants in this study were not diagnosed with cognitive disorders, they are vulnerable to age-related cognitive decline, which could be a part of the natural aging process. The observed effects of 5-HTP supplementation on cognitive function and mood outcomes may reflect preventive or maintaining benefits in this population without overt impairment. Additionally, both validated subjective questionnaires and objective blood biomarker assessment tools were applied to assess cognitive function and to explore the potential mechanism in this study. Lastly, the dietary intake throughout the intervention was well controlled as evidenced by a maintenance of nutrient intake, which can further support the improvements in cognitive function and mood by 5-HTP supplementation.

However, there are some limitations to consider. Firstly, the effect of 5-HTP on cognitive function was the secondary outcome in this study. Furthermore, due to the lack of preliminary data, the power calculation, although performed liberally, may remain inadequate for the cognitive function investigation. Further studies with a larger sample size are warranted to confirm the efficacy of 5-HTP supplementation and explore subgroup effects, such as those based on gender, narrower age groups, and different health conditions. As participants were relatively healthy in this study, it is hard to generalize our findings to those who are diagnosed with cognitive disorders. Further studies are warranted to establish the MCID range specific to cognitively healthy older adults. The daily dietary intake, sleep conditions, and cardiometabolic profiles were maintained during the study; however, besides these factors, various lifestyle factors such as physical activity and social engagement, which could also potentially influence the outcomes, were not measured. Therefore, future studies may benefit from a more comprehensive lifestyle assessment to better interpret the effect of the dietary intervention. Although validated tools were applied, assessments of cognitive function and mood mainly relied on self-reporting, which could lead to reporting bias. Additionally, though different versions of MoCA were used at every visit, the repeated administration of the MoCA would inevitably lead to practice effects. Moreover, the large variability of cognitive function-related biomarker measurements limits the interpretation of the results. Lastly, the blood serotonin levels may not fully reflect the central serotonin levels, which are directly implicated in neuropsychiatric and neurodegenerative disorders [65], and the employment of neuroimaging or cerebrospinal fluid analysis can be considered to further investigate the mechanistic insight.

## 5. Conclusions

In conclusion, 5-HTP supplementation demonstrated potential in improving cognitive function and alleviating depression symptoms through the regulation of the serotonergic system in Singaporean older adults. This finding highlights the promising effect of 5-HTP as an accessible complementary supplementation to promote cognitive function and mental health in the aging population, though further studies with a larger sample size and more sensitive tools are warranted to confirm its efficacy in the long term and in broader populations.

## Figures and Tables

**Table 1:** Baseline characteristics of the 5-HTP and control group.

	5-HTP	Control	*p* Value
	Mean ± SD	Mean ± SD	
Age (years) ^a^	66 ± 3	67 ± 4	0.4461
Gender (F/M)	8/7	7/8	
Race (Chinese/Indian/Caucasian)	15/0/0	12/1/2	
Education Level			
Secondary (%)	27%	40%	
Tertiary (%)	47%	33%	
University and above (%)	27%	27%	
Medical Condition			
High blood pressure (%)	13%	27%	
High blood cholesterol (%)	20%	60%	
Osteoporosis (%)	13%	13%	
Osteopenia (%)	7%	13%	
Anthropometrics			
Weight (kg) ^a^	57.3 ± 11.3	61.5 ± 16.7	0.4280
BMI (kg/m^2^) ^b^	21.8 ± 2.6	23.8 ± 5.5	0.3245
Waist Circumference (cm) ^a^	80.9 ± 10.2	85.4 ± 14.3	0.3400
Blood pressure			
Systolic (mmHg) ^b^	120 ± 23	126 ± 19	0.1702
Diastolic (mmHg) ^a^	72 ± 14	79 ± 11	0.1426

BMI: Body mass index, SD: Standard deviation. ^a^ The data were analyzed by the Welch’s *t* test. ^b^ The data were analyzed by the Mann–Whitney *t* test.

**Table 2:** MoCA scores of the 5-HTP and control group.

		5-HTP	Control	*p* Value	
		Mean ± SD	Mean ± SD	Time	Group × Time
Total Score	Week 0	26.6 ± 1.4	26.4 ± 1.8	0.0007	0.9343
	Week 4	26.4 ± 1.2	26.2 ± 1.9		
	Week 8	27.2 ± 1.9	27.5 ± 1.7		
	Week 12	27.6 ± 1.4 *	27.3 ± 2.4		
Visuospatial/ Executive	Week 0	4.6 ± 0.5	4.7 ± 0.6	0.7582	0.9363
	Week 4	4.6 ± 0.6	4.7 ± 0.5		
	Week 8	4.7 ± 0.5	4.9 ± 0.4		
	Week 12	4.8 ± 0.6	4.9 ± 0.4		
Naming	Week 0	2.4 ± 0.5	2.6 ± 0.5	<0.0001	0.4317
	Week 4	2.9 ± 0.4	3.0 ± 0.0 *		
	Week 8	3.0 ± 0.0 *	3.0 ± 0.0 *		
	Week 12	3.0 ± 0.0 *	3.0 ± 0.0 *		
Attention	Week 0	6.0 ± 0	5.7 ± 0.5	0.1448	0.0880
	Week 4	5.9 ± 0.3	5.8 ± 0.4		
	Week 8	5.4 ± 1.1	5.7 ± 0.5		
	Week 12	5.9 ± 0.3	5.7 ± 0.5		
Language	Week 0	1.4 ± 0.7	1.6 ± 1.1	0.0003	0.7277
	Week 4	1.1 ± 0.6	1.3 ± 0.7		
	Week 8	1.9 ± 0.7	2.2 ± 0.7		
	Week 12	1.8 ± 0.8	1.7 ± 1.1		
Abstraction	Week 0	1.6 ± 0.6	1.8 ± 0.4	0.0070	0.8541
	Week 4	1.5 ± 0.5	1.6 ± 0.5		
	Week 8	1.9 ± 0.3	1.9 ± 0.3		
	Week 12	1.8 ± 0.4	1.8 ± 0.4		
Delayed Recall	Week 0	4.6 ± 0.6	4.2 ± 0.9	0.2386	0.5258
	Week 4	4.6 ± 0.6	3.9 ± 1.2		
	Week 8	4.3 ± 0.9	3.8 ± 1.5		
	Week 12	4.5 ± 0.8	4.3 ± 1.0		
Orientation	Week 0	5.9 ± 0.3	5.8 ± 0.4	0.2026	0.2211
	Week 4	5.7 ± 0.5	5.9 ± 0.4		
	Week 8	5.9 ± 0.3	5.9 ± 0.3		
	Week 12	5.9 ± 0.4	5.9 ± 0.3		

MoCA: Montreal Cognitive Assessment, SD: Standard Deviation. The data were analyzed by repeated-measures two-way ANOVA. * Denoted the significant difference within groups compared to the baseline visit using two-way repeated measure ANOVA: Tukey’s multiple comparisons test, *p* < 0.05.

**Table 3:** Levels of cognitive function-related biomarkers of the 5-HTP and the control group.

	5-HTP			Control			*p* Value	
	Week 0	Week 12	Week 12–0 Change	Week 0	Week 12	Week 12–0 Change	Time	Group × Time
Aβ40 (pg/mL)	14.4 ± 23.4	13.8 ± 25.5	−0.6 ± 3.2	9.8 ± 8.5	7.8 ± 8.8	−2.0 ± 4.3	0.2871	0.5523
Aβ42 (pg/mL)	7.5 ± 7.5	6.9 ± 7.2	−0.6 ± 0.8	6.7 ± 9.1	4.9 ± 6.6	−1.8 ± 2.6	0.0786	0.3634
Aβ42/Aβ40	1.2 ± 1.2	18.9 ± 41.0	17.7 ± 40.1	0.9 ± 0.8	7.2 ± 14.8	6.3 ± 15.0	0.2045	0.5302
GABA (ng/mL)	2.7 ± 0.7	2.8 ± 0.8	0.1 ± 0.3	2.5 ± 0.8	2.6 ± 0.8	0.1 ± 0.5	0.1912	0.7411
Serotonin (ng/mL)	173.7 ± 81.2	219.6 ± 73.1 *	45.8 ± 64.8 ^a^	209.5 ± 87.0	185.4 ± 95.6	−20.2 ± 75.3 ^b^	0.3432	0.0197

Aβ; Amyloid beta, GABA: Gamma-aminobutyric acid. Data are shown as mean ± standard deviation. The data were analyzed by Mann–Whitney t test and repeated-measures two-way ANOVA. ^a,b^ Denoted the significant difference between groups of changes, *p* < 0.05. * Denoted the significant difference within groups compared to the baseline visit using two-way repeated measure ANOVA: Tukey’s multiple comparisons test, *p* < 0.05.

**Table 4:** GDS and GAI scores of the 5-HTP and the control group.

		5-HTP	Control	*p* Value	
		Mean ± SD	Mean ± SD	Time	Group × Time
GDS	Week 0	1.2 ± 1.7	1.4 ± 2.3	0.0564	0.3851
	Week 4	0.8 ± 1.3	1.3 ± 2.1		
	Week 8	0.7 ± 1.2 *	0.7 ± 1.2		
	Week 12	0.6 ± 1.4	1.3 ± 2.5		
GAI	Week 0	1.7 ± 4.6	1.3 ± 2.1	0.5615	0.1676
	Week 4	0.6 ± 1.5	2.1 ± 5.0		
	Week 8	0.6 ± 2.3	1.6 ± 4.9		
	Week 12	1.1 ± 3.9	0.9 ± 2.6		

GDS: Geriatric Depression Scale; GAI: Geriatric Anxiety Inventory; SD: Standard Deviation. The data were analyzed by repeated-measures two-way ANOVA. * Denotes the significant difference within groups compared to the baseline visit using two-way repeated-measures ANOVA: Dunnett’s multiple comparisons test, *p* < 0.05.

## Data Availability

The original contributions presented in this study are included in the article. Further inquiries can be directed to the corresponding author.

## Supplementary Materials

The following supporting information can be downloaded at: https://www.mdpi.com/article/10.3390/nu17172773/s1, Table S1: Nutrients intake of the 5-HTP group and the control group.
