# Supplementary material for: The Impact of 5-Hydroxytryptophan Supplementation on Cognitive Function and Mood in Singapore Older Adults: A Randomized Controlled Trial
Source: Nutrients. 2025 Aug 27;17(17):2773. doi: 10.3390/nu17172773 (PMC12430700; doi:10.3390/nu17172773)
Supplement: Supplementary file 1 [file nutrients-17-02773-s001.zip › nutrients-3759165-supplementary.pdf]

Supplementary Materials

Table S1. Nutrients intake of the 5-HTP group and the control group

		5-HTP	Control	*P* value	
		Mean ± SD	Mean ± SD	Time	Group × Time
Energy (kcal)	Week 0	1712 ± 523	1580 ± 384	0.6051	0.3015
	Week 4	1659 ± 513	1625 ±406		
	Week 8	1796 ± 442	1577 ± 531		
	Week 12	1588 ± 447	1692 ± 458		
Carbohydrate (g/d)	Week 0	197 ± 60	160 ± 36	0.5832	0.7956
	Week 4	191 ± 63	175 ± 45		
	Week 8	201 ± 69	178 ± 56		
	Week 12	188 ± 55	165 ± 46		
Protein (g/ d)	Week 0	77 ± 27	69 ± 23	0.9701	0.1530
	Week 4	69 ± 29	75 ± 27		
	Week 8	76 ± 24	71 ± 29		
	Week 12	70 ± 27	75 ± 22		
Fat (g/d)	Week 0	69 ± 30	66 ± 27	0.9410	0.2077
	Week 4	64 ± 26	66 ± 25		
	Week 8	71 ± 25	59 ± 29		
	Week 12	63 ± 19	68 ± 29		
Fiber (g/d)	Week 0	19.2 ± 4.9	15.9 ± 5.7	0.4449	0.4016
	Week 4	18.9 ± 6.4	18.1 ± 6.9		
	Week 8	22.1 ± 8.7	17.5 ± 6.8		
	Week 12	18.5 ± 6.4	18.0 ± 6.6		
Tryptophan (g/d)	Week 0	0.9 ± 0.3	0.8 ± 0.3	0.8982	0.1939
	Week 4	0.8 ± 0.4	0.9 ± 0.3		
	Week 8	0.9 ± 0.3	0.8 ± 0.4		
	Week 12	0.8 ± 0.3	0.9 ± 0.3		
LNAA (g/d)	Week 0	18.9 ± 7.0	17.0 ± 5.8	0.9203	0.2311
	Week 4	17.4 ± 7.4	18.6 ± 7.4		
	Week 8	19.0 ± 6.5	17.8 ± 7.5		
	Week 12	17.2 ± 6.6	18.4 ± 5.6		
EPA (g/d)	Week 0	0.2 ± 0.2	0.2 ± 0.4	0.5785	0.3957
	Week 4	0.1 ± 0.1	0.2 ± 0.2		
	Week 8	0.2 ± 0.2	0.1 ± 0.1		
	Week 12	0.2 ± 0.2	0.1 ± 0.1		
DHA (g/d)	Week 0	0.4 ± 0.5	0.5 ± 0.7	0.7993	0.5468
	Week 4	0.3 ± 0.2	0.4 ± 0.4		
	Week 8	0.4 ± 0.5	0.3 ± 0.3		
	Week 12	0.4 ± 0.4	0.3 ± 0.2		

Sodium (g/d)	Week 0	1.8 ± 0.7	2.3 ± 1.1	0.9970	0.3858
	Week 4	2.0 ± 0.7	2.2 ± 1.1		
	Week 8	2.1 ± 0.7	2.1 ± 1.0		
	Week 12	1.8 ± 0.6	2.3 ± 0.9		
Potassium (mg/d)	Week 0	2533.0 ± 1070.6	2429.5 ± 845.7	0.1357	0.3801
	Week 4	2441.2 ± 1007.5	2554.6 ± 676.6		
	Week 8	2656.4 ± 1131.1	2317.4 ± 643.6		
	Week 12	2263.6 ± 1013.6	2201.6 ± 592.5		
Calcium (mg/d)	Week 0	535.3 ± 199.5	575.3 ± 246.6	0.6127	0.4354
	Week 4	540.4 ± 206.3	526.7 ± 220.9		
	Week 8	617.3 ± 249.4	534.5 ± 234.7		
	Week 12	568.3 ± 246.4	588.9 ± 243.5		
Magnesium (mg/d)	Week 0	327.5 ± 154.3	303.2 ± 77.7	0.7875	0.4437
	Week 4	310.6 ± 153.1	312.7 ± 95.0		
	Week 8	353.7 ± 195.6	296.0 ± 131.3		
	Week 12	300.1 ± 140.6	310.2 ± 111.6		
Vitamin C (mg/d)	Week 0	117.3 ± 84.6	86.3 ± 53.5	0.2358	0.7815
	Week 4	108.2 ± 69.3	93.7 ± 42.4		
	Week 8	152.3 ± 103.6	110.3 ± 102.5		
	Week 12	101.4 ± 50.9	93.8 ± 85.9		
Vitamin B6 (mg/d)	Week 0	2.6 ± 3.2	1.8 ± 0.7	0.7277	0.2566
	Week 4	1.9 ± 1.1	2.0 ± 0.4		
	Week 8	2.3 ± 1.2	2.0 ± 0.7		
	Week 12	1.9 ± 1.2	2.3 ± 1.2		
Folate (μg/d)	Week 0	287.1 ± 118.8	223.0 ± 77.8	0.0964	0.0820
	Week 4	241.4 ± 79.5	288.1 ± 87.1		
	Week 8	337.9 ± 152.8	278.5 ± 132.8		
	Week 12	247.7 ± 89.2	251.2 ± 87.0		
Vitamin B12 (μg/d)	Week 0	3.5 ± 2.4	3.5 ± 2.1	0.3977	0.4934
	Week 4	3.2 ± 1.9	4.0 ± 2.1		
	Week 8	4.8 ± 2.4	3.1 ± 2.2		
	Week 12	4.9 ± 4.7	3.8 ± 2.6		
Vitamin E (mg/d)	Week 0	2.4 ± 2.6	2.8 ± 2.9	0.2967	0.7515
	Week 4	2.0 ± 1.3	2.0 ± 1.5		
	Week 8	2.0 ± 1.4	1.7 ± 1.7		
	Week 12	2.2 ± 1.2	1.8 ± 1.2		
Vitamin D (μg/d)	Week 0	0.4 ± 0.3	2.1 ± 2.4	0.5354	0.0745
	Week 4	1.2 ± 2.7	2.6 ± 4.8		
	Week 8	1.2 ± 2.0	1.0 ± 1.0		
	Week 12	2.2 ± 5.1	1.0 ± 1.0		

LNAA; Large natural amino acid, EPA; Eicosapentaenoic acid; DHA; Docosahexaenoic acid, SD; Standard deviation. The data were analyzed by repeated measures two-way ANOVA.
